# The distribution of toxic metals in the human retina and optic nerve head: Implications for age-related macular degeneration

**DOI:** 10.1371/journal.pone.0241054

**Published:** 2020-10-29

**Authors:** Roger Pamphlett, Svetlana Cherepanoff, Lay Khoon Too, Stephen Kum Jew, Philip A. Doble, David P. Bishop

**Affiliations:** 1 Discipline of Pathology, School of Medical Sciences, Brain and Mind Centre, The University of Sydney, Sydney, New South Wales, Australia; 2 Department of Neuropathology, Royal Prince Alfred Hospital, Sydney, New South Wales, Australia; 3 Sydpath, St Vincent’s Hospital, Sydney, Australia; 4 St Vincent’s Clinical School, University of New South Wales, Sydney, Australia; 5 Faculty of Medicine and Health, The University of Sydney, Sydney, New South Wales, Australia; 6 Elemental Bio-Imaging Facility, School of Mathematical and Physical Sciences, University of Technology Sydney, Sydney, New South Wales, Australia; Chinese Academy of Sciences, CHINA

## Abstract

**Objective:**

Toxic metals are suspected to play a role in the pathogenesis of age-related macular degeneration. However, difficulties in detecting the presence of multiple toxic metals within the intact human retina, and in separating primary metal toxicity from the secondary uptake of metals in damaged tissue, have hindered progress in this field. We therefore looked for the presence of several toxic metals in the posterior segment of normal adult eyes using elemental bioimaging.

**Methods:**

Paraffin sections of the posterior segment of the eye from seven tissue donors (age range 54–74 years) to an eye bank were examined for toxic metals *in situ* using laser ablation-inductively coupled plasma-mass spectrometry, a technique that detects multiple elements in tissues, as well as the histochemical technique of autometallography that demonstrates inorganic mercury, silver, and bismuth. No donor had a visual impairment, and no significant retinal abnormalities were seen on *post mortem* fundoscopy and histology.

**Results:**

Metals found by laser ablation-inductively coupled plasma-mass spectrometry in the retinal pigment epithelium and choriocapillaris were lead (n = 7), nickel (n = 7), iron (n = 7), cadmium (n = 6), mercury (n = 6), bismuth (n = 5), aluminium (n = 3), and silver (n = 1). In the neural retina, mercury was present in six samples, and iron in one. Metals detected in the optic nerve head were iron (N = 7), mercury (N = 7), nickel (N = 4), and aluminium (N = 1). No gold or chromium was seen. Autometallography demonstrated probable inorganic mercury in the retinal pigment epithelium of one donor.

**Conclusion:**

Several toxic metals are taken up by the human retina and optic nerve head. Injury to the retinal pigment epithelium from toxic metals could damage the neuroprotective functions of the retinal pigment epithelium and allow toxic metals to enter the outer neural retina. These findings support the hypothesis that accumulations of toxic metals in the retina could contribute to the pathogenesis of age-related macular degeneration.

## Introduction

Age-related macular degeneration (AMD) is a common cause of visual impairment in people over the age of 55 years [1]. The pathology of AMD involves degenerative changes to the retinal pigment epithelium (RPE), the adjacent Bruch’s membrane, the choriocapillaris (choroid), and the neurosensory (‘neural’) retina [1]. The RPE is affected early and severely in AMD, and because its many functions protect the neural retina from damage [2] interest has arisen in finding agents that could injure the RPE. One proposal is that toxic metals from environmental sources accumulate in the RPE over time, until their concentrations reach a tipping point that impairs the function of RPE cells, with subsequent injury to the photoreceptors of the adjacent outer neural retina [3–7].

Toxic metals have previously been detected in the human RPE, choriocapillaris and neural retina [3–7]. In four donors (aged 76–90 years, without clinicopathological details) X-ray microanalysis of 3x3 mm samples of RPE detected iron in melanosomes of all four donors, aluminium in three, and mercury in one [3]. Lead was found to be higher, using atomic absorption spectrophotometry, in the combined RPE/neural retina than in the choriocapillaris from eight eye donors (aged 19–90 years, without ocular histology) [4]. When the eyes of 16 donors (aged 62–94 years), some of which contained macular drusen, were examined with inductively coupled plasma-mass spectrometry, the RPE/choriocapillaris of all samples contained lead and cadmium; the neural retina contained cadmium in 100% and lead in 30% [5]. In 22 donors of normal eyes (aged 16 to 87 years) inductively coupled plasma-mass spectrometry and atomic absorption spectrophotometry showed that average cadmium levels were high in the RPE in those aged <55 years, and high in the RPE, choriocapillaris and neural retina in those aged 55 years and over [6]. A comparison of toxic metal levels in 39 control (mean age 83 years) eyes and 51 AMD (mean age 80 years) eyes, using inductively coupled plasma-mass spectrometry, showed that average levels of lead, cadmium, chromium, arsenic and nickel were higher in both the RPE/choriocapillaris and neural retina in the AMD group; in the control group, raised levels of metals were present only occasionally in the RPE/choriocapillaris (nickel in three, cadmium in two, and lead and chromium in one each), while in only one neural retina was nickel high [7].

In all these previous studies, individual retinal layers, or combinations of RPE, choriocapillaris and neural retina layers, had to be separated before study, and in those using inductively coupled plasma-mass spectrometry or atomic absorption spectrophotometry, samples had to be digested before analyses, so the cellular locations of metals could not be confirmed. In no studies have the presence of multiple toxic metals been able to be assessed within structurally intact human retina samples. We therefore looked for toxic metals in the normal retina *in situ* using two techniques, laser ablation-inductively coupled plasma-mass spectrometry (LA-ICP-MS), which detects multiple elements simultaneously in tissues, and autometallography which demonstrates intracellular inorganic mercury, silver, and bismuth. These elemental analyses enabled us to assess the presence of toxic metals within the normal aging retina, so that primary metal accumulation (that could predispose to AMD) could be distinguished from the secondary uptake of metals by retinal cells that had been damaged by AMD [1, 7–9].

## Materials and methods

### Ethics statement

This study (USYD2014-792) was approved by the University of Sydney Human Research Ethics Committee and was conducted according to the principles expressed in the Declaration of Helsinki. Donors gave signed consent for their eye tissue to be used for research purposes.

### Tissue samples

Eyes for this study were removed at death for corneal transplantation in 2015 and 2016, and consisted of posterior globes, not needed for corneal transplantation, that were subjected to *post mortem* fundoscopy [10], then dissected in the horizontal plane, fixed in formalin, and processed routinely for paraffin embedding. Inclusion criteria for the study were: age between 50–80 years, no history of retinal disease, no retinal abnormalities on *post mortem* fundoscopy assessed by at least two ophthalmologists, and no significant histological retinal abnormalities assessed by at least two pathologists [11–13]. The 7 donors (4 male, 3 female) had a mean age of 61 years (SD 7 years, range 54–74 years), a mean *post mortem* delay of 19 hours (SD 5 hours, range 12–24 hours), and had histories of esophageal cancer (n = 2), lung cancer, rectal cancer and hypertension, ischemic heart disease, atrial fibrillation, and cardiomyopathy.

### Laser ablation-inductively coupled plasma-mass spectrometry (LA-ICP-MS)

Seven-μm paraffin sections were cut with a Feather S35 stainless steel disposable microtome blade and deparaffinised. Sections were subjected to LA-ICP-MS for iron (Fe), phosphorus (P, to assess nuclear density [3]), mercury (Hg), silver (Ag), aluminium (Al), gold (Au), bismuth (Bi), cadmium (Cd), chromium (Cr), nickel (Ni) and lead (Pb). Analyses were carried out on an LSX-213 G2+ laser (Teledyne Cetac) hyphenated to an Agilent Technologies 8900 ICP-MS, with argon used as the carrier gas. LA-ICP-MS conditions were optimised on NIST 612 Trace Element in Glass CRM and the sample was ablated with a 50 μm spot size and a scan speed of 100 μm/s at a frequency of 20 Hz. The data were collated into a single image file using in-house developed software and visualised using FIJI. Based on relative abundance, the amounts of elements in tissues were classified qualitatively as being either not detected (-), sparse (+) or abundant (++).

### Autometallography

Seven-μm paraffin sections were deparaffinised and stained for inorganic mercury, silver or bismuth bound to sulphide or selenide using silver nitrate autometallography, which represents the presence of these metals as black grains, since the metal sulphides or selenides catalyze the reduction of silver ions to visible metallic silver [14]. Briefly, sections were placed in physical developer containing 50% gum arabic, citrate buffer, hydroquinone and silver nitrate at 26°C for 80 min in the dark then washed in 5% sodium thiosulphate to remove unbound silver. Sections were counterstained with mercury-free hematoxylin and viewed with bright-field microscopy. Each staining run included a control section of mouse spinal cord where motor neuron cell bodies contained mercury following an intraperitoneal injection of mercuric chloride [15]. Adjacent sections were stained with hematoxylin only as a control for the autometallography/hematoxylin sections.

## Results

### Eye histology

The normal histology of the retina and optic nerve head of the seven donors can be seen in S1–S7 Figs.

### Laser ablation-inductively coupled plasma-mass spectrometry

**Fig 1:**
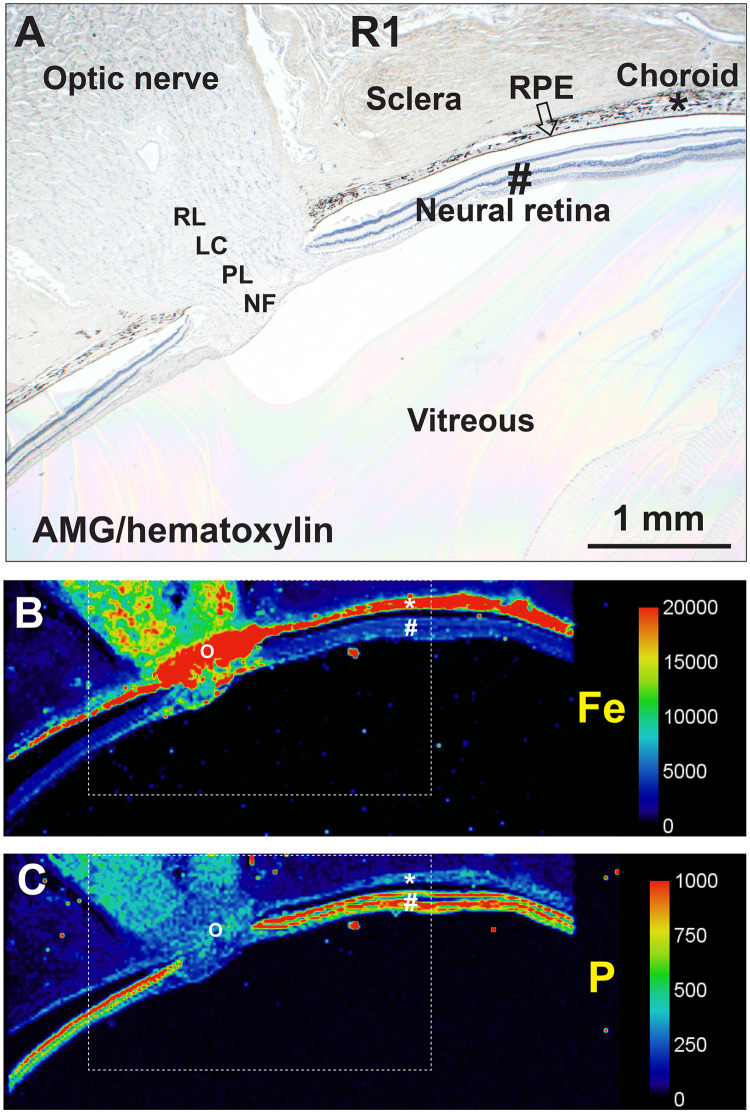
Identification of the RPE/choriocapillaris and neural retina. (**A**) A low-power histological view of the sample, stained with autometallography/hematoxylin (AMG/H), shows the sclera, choriocapillaris (*), RPE (arrow), neural retina (#), and optic nerve head. The RPE is artefactually separated from the neural retina. The area corresponding to this histological image is shown in the dashed boxes in the LA-ICP-MS images below. Optic nerve head anatomical regions are NF: superficial nerve fibre layer, PL: prelaminar region, LC: lamina cribrosa, and RL: retrolaminar region. (**B**) LA-ICP-MS of an adjacent section shows iron (Fe) in the RPE/choriocapillaris (*), and in the optic nerve head (O). (**C**) LA-ICP-MS for phosphorus shows the high cellularity of the neural retina (#), and the lower cellularity of the RPE (*) and optic nerve head (O). The separation of the RPE and the neural retina, and a smaller separation within the neural retina (to the right of the #), can be seen.

In all samples, iron was visible in the RPE/choriocapillaris, and phosphorus in the highly-cellular neural retina, so these elements were used as markers for these regions (Fig 1). Common artefactual separation of the RPE and neural retina (Fig 1), and of the RPE and choriocapillaris, aided identification of the different cell layers by LA-ICP-MS.

#### RPE/choriocapillaris

**Fig 2:**
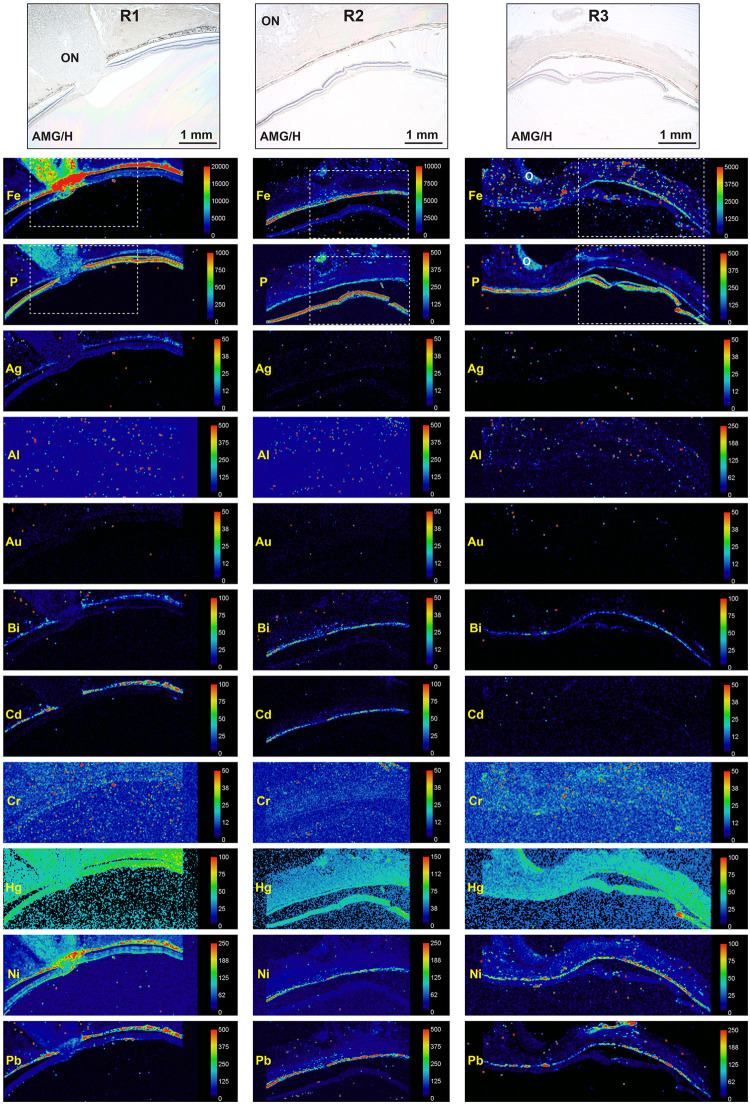
LA-ICP-MS of three eye samples. The histological images at the top of the figure, stained with autometallography/hematoxylin (AMG/H), are from sections adjacent to the LA-ICP-MS samples. The areas corresponding to these histological images are shown in the dashed boxes in the iron (Fe) and phosphorus (P) LA-ICP-MS images. The toxic metals sampled are presented below in alphabetical order. The location of the toxic metals in the eye can be assessed by reference to the iron image (high levels in the RPE/choriocapillaris) and to the phosphorus image (high levels in the neural retina, low levels in the RPE/choriocapillaris). The optic nerve head (O) is not included in the R3 AMG image. Scale = counts per second (proportional to abundance).

**Fig 3:**
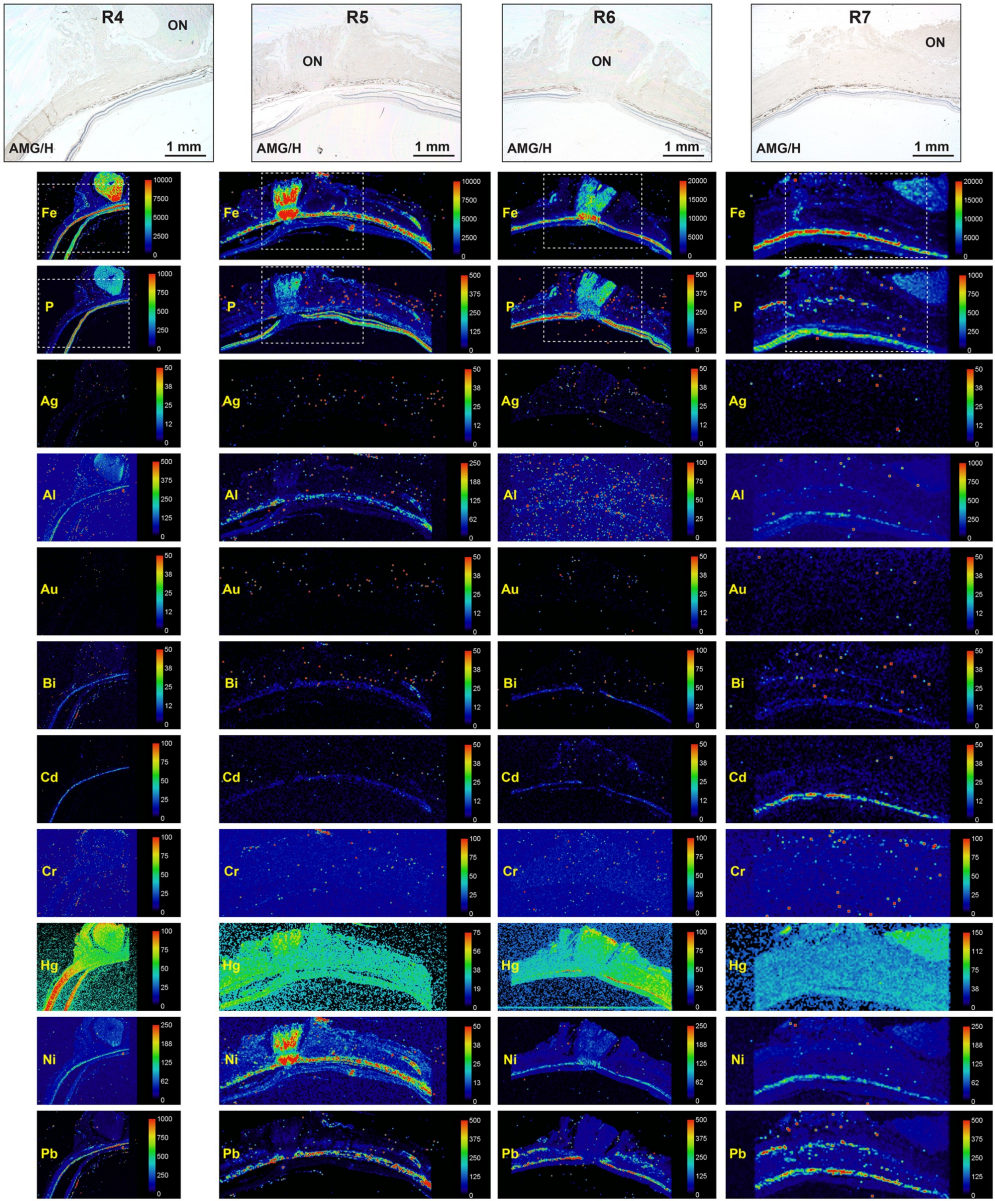
LA-ICP-MS of four eye samples. The histological images at the top of the figure, stained with autometallography/hematoxylin (AMG/H), are from sections adjacent to the LA-ICP-MS samples. The areas corresponding to these histological images are shown in the dashed boxes in the iron (Fe) and phosphorus (P) LA-ICP-MS images. The toxic metals sampled are presented below in alphabetical order. The location of the toxic metals in the eye can be assessed by reference to the iron image (high levels in the RPE/choriocapillaris) and to the phosphorus image (high levels in the neural retina, low levels in the RPE/choriocapillaris). Scale = counts per second (proportional to abundance).

The toxic metals detected most commonly in the RPE/choriocapillaris by LA-ICP-MS were lead (n = 7), nickel (n = 7), cadmium (n = 6), mercury (n = 6), and bismuth (n = 5), followed by aluminium (n = 3) and silver (n = 1) (Figs 2 and 3, Table 1). The sample was ablated with a 50 μm spot size, so the neural retina and RPE/choriocapillaris could be separated from each other. Distinguishing the RPE (a single layer of cells, about 10 μm thick) from the closely adjacent choriocapillaris was difficult in most samples, apart from in R2, R4 and R6 where thin continuous signals were likely to have come from the RPE alone.

**Table 1:** Elements found by LA-ICP-MS in the posterior segment of seven adult eyes.

	Site	Fe	P	Ag	Al	Au	Bi	Cd	Cr	Hg	Ni	Pb
R1	RPE/CHO	++	+	+	-	-	+	++	-	+	++	++
	Neural retina	-	++	-	-	-	-	-	-	+	-	-
	Optic nerve	++	+	-	-	-	-	-	-	+	+	-
R2	RPE/CHO	++	+	-	-	-	+	+	-	+	++	++
	Neural retina	-	++	-	-	-	-	-	-	+	-	-
	Optic nerve	+	++	-	-	-	-	-	-	+	-	-
R3	RPE/CHO	+	-	-	-	-	+	-	-	+	++	++
	Neural retina	-	++	-	-	-	-	-	-	+	-	-
	Optic nerve	+	+	-	-	-	-	-	-	+	-	-
R4	RPE/CHO	++	+	-	+	-	+	+	-	++	+	++
	Neural retina	+	++	-	-	-	-	-	-	++	-	-
	Optic n	+	+	-	+	-	-	-	-	+	+	-
R5	RPE/CHO	++	+	-	+	-	-	+	-	+	++	++
	Neural retina	-	++	-	-	-	-	-	-	+	-	-
	Optic nerve	++	+	-	-	-	-	-	-	+	++	-
R6	RPE/CHO	++	-	-	-	-	+	+	-	+	+	++
	Neural retina	-	++	-	-	-	-	-	-	+	-	-
	Optic nerve	++	+	-	-	-	-	-	-	+	+	-
R7	RPE/CHO	++	+	-	+	-	-	++	-	-	+	++
	Neural retina	-	++	-	-	-	-	-	-	-	-	-
	Optic nerve	+	+	-	-	-	-	-	-	+	-	-

R: donor identification number, RPE/CHO: retinal pigment epithelium and choriocapillaris,—not detected, + sparse, ++ abundant.

#### Neural retina

In the neural retina, mercury was seen at high levels in one sample (R4), and at low levels in five, while iron was seen in one sample (R4) (Fig 3, Table 1).

#### Optic nerve head

The metals observed in the optic nerve head were iron (N = 7), mercury (N = 7), nickel (N = 4), and aluminium (N = 1) (Figs 2 and 3, Table 1). Iron and nickel were seen predominantly in the prelaminar region and lamina cribrosa of the optic nerve head. Unlike the choriocapillaris, no collections of red blood cells were seen in the optic nerve heads (S1–S7 Figs), indicating that the metals detected here were in the tissue and not within red blood cells.

### Autometallography

**Fig 4:**
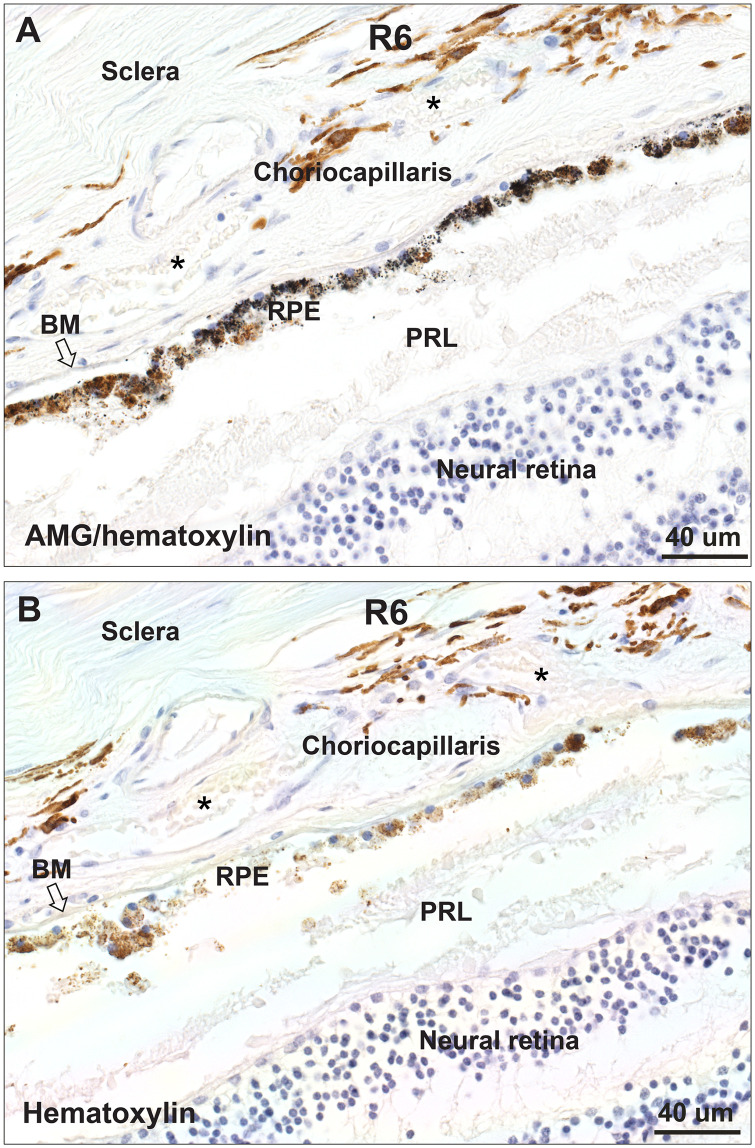
Autometallography of the retina and choriocapillaris of R6. (**A**) In the section stained with autometallography/hematoxylin, small black autometallographic grains are seen in most retinal pigment epithelium (RPE) cells. Brown-coloured melanosomes can also be seen in some of these cells. No black grains are seen in the pigmented cells of the choriocapillaris, in the retina, or in the sclera. Red blood cells (*, pale yellow) are seen in capillaries. The photoreceptor layer (PRL) of the outer neural retina is artefactually fragmented. (**B**) In an adjacent section stained with hematoxylin only, normal brown melanosomes, but no black grains, are present in the RPE. Red blood cells (*, pale yellow) are seen in capillaries. BM: Bruch’s membrane.

Small black autometallographic grains were present in RPE cells in R6, which could be distinguished from the normal brown melanosomes in RPE cells seen on adjacent hematoxylin-only stained sections (Fig 4). No autometallography was seen in the nearby pigmented choriocapillaris cells. The autometallography in R6 corresponded to *mercury* detected on LA-ICP-MS in this RPE, so although some bismuth was also present here (see below) we assumed the autometallography detected mercury (Fig 3). This suggests the LA-ICP-MS mercury in the R4 RPE, and in a more widespread distribution in other samples, is in the organic form, since autometallography demonstrates only inorganic mercury bound to sulphides and selenides [14]. A small amount of *bismuth* was present in the R6 RPE on LA-ICP-MS, but in this, and other samples with stronger bismuth LA-ICP-MS signals, no autometallography was seen, suggesting bismuth in the retina was not in the inorganic form that is recognised by autometallography [16]. Inorganic *silver* was not detected on autometallography in the RPE of R1, where a small amount of LA-ICP-MS silver was seen (Fig 2).

## Discussion

Key findings in this study are that different combinations of toxic metals can be found in the structurally intact RPE/choriocapillaris, neural retina, and optic nerve head of adults who have no clinical or pathological features of AMD. The location of these metals in human eyes closely resembles that found in monkeys exposed to large doses of mercury vapor [17].

The RPE and choriocapillaris took up toxic metals most avidly in our study, probably because metals can pass through fenestrated capillaries in the choriocapillaris [2] and are then taken up actively by RPE cells [18]. Of the metals we found in the RPE/choriocapillaris, cadmium, mercury and lead are the three most commonly associated with human toxicity [19]. Generation of reactive oxygen species and inflammation have been described as an effect of cadmium, mercury, and lead, as well as of iron, nickel, aluminium, and silver [20–23]. Other toxic mechanisms of cadmium, mercury, and lead are immunotoxicity, apoptosis, membrane and organelle damage, genotoxicity, and alterations of the epigenome [19], all of which have been implicated in the pathogenesis of AMD [24, 25]. The location of multiple toxic metals within the RPE/choriocapillaris is relevant to the finding of adverse synergistic interactions between toxic metals [21, 22], and the generation of reactive oxygen species is one of the mechanisms most augmented by metal-metal interactions [22].

Other potentially toxic metals in the RPE/choriocapillaris, ie, iron, nickel, aluminium, bismuth and silver, were seen in our retinal samples. *Iron*, although an essential element, can become toxic if present in high concentrations in tissues, because as free iron it can produce oxygen free radicals via the Fenton reaction [26]. *Nickel* is only a mild activator of reactive oxygen species, but can deplete glutathione levels, bind to sulfhydryl groups of proteins, and act as an immunotoxin and genotoxin [20]. *Aluminium* can cross-link proteins and is thought to generate oxygen radical species [22, 27]. *Silver* in nanoparticles has been reported to induce oxidative stress in several animal experiments [28]. *Bismuth* can cause an encephalopathy, and appears to target astrocyte metabolism, but does not appear to cause oxidative stress [29]. Bismuth is deposited in tissue adjacent to blood vessels with fenestrated endothelium [30], the same type of endothelium as in the choriocapillaris.

Several lines of evidence suggest toxic metals play a role in AMD. Many of the toxic metals detected in our study can precipitate oxidative stress and inflammation, which are the most frequently cited mechanisms thought to underlie AMD [24, 25, 31]. AMD is more common in people who smoke tobacco [1], which contains cadmium, lead, mercury and nickel, and is more common in people who have been exposed to cadmium, mercury or lead [32, 33]. Mercury is taken up by the retina of mercury-exposed mice [34, 35], rats [36], and monkeys [17, 36, 37]. Exposure to mercury affects human vision [32, 38, 39], and people with higher blood levels of mercury from amalgam fillings have more retinal thinning on optical coherence tomography than controls [40]. Mercury damages human RPE cells in culture [41], and inorganic mercury is taken up in RPE cells via amino acid transporters [18].

The fact that AMD appears only later in life may be because toxic metals need to accumulate in the retina over long periods of time before a tipping point of metal concentration is reached that damages the RPE. Uptake of metals in the retina could start early in life, since exposure to mercury in both pregnant monkeys [37] and pregnant mice [35] results in mercury being laid down in the fetal retina. Cell turnover in the RPE is slow or absent in the normal adult retina [42], so continuous or repeated exposures to toxic metals are likely to result in accumulation of these xenobiotics over time. Another possible reason for the late appearance of AMD is that most of our eye samples appeared to contain organic mercury; human exposure to organic mercury from consuming mercury-containing seafood is common [43, 44], and it takes years for organic mercury to be converted in cells to toxic inorganic mercury [45].

Total mercury detected on LA-ICP-MS was widespread in the posterior segment of most eyes, but inorganic mercury was seen in the RPE in only one eye. This suggests most of our donors were exposed to organic mercury, probably from seafood consumption since over 90% of Australians report eating seafood regularly [44]. This implies that organic mercury can be cleared from cells in the posterior segment of the eye apart from those in the RPE, where mercury is bound to melanosomes [3]. This fits with the finding in fetal monkeys, exposed to mercury during gestation, that shortly after birth widespread retinal mercury was present, but years later mercury was seen only in the RPE [37].

Previous reports have described toxic metals in the human choriocapillaris as well as in the RPE [5–7], and toxic metals were often detected in the choriocapillaris of our samples. Although the role of the choriocapillaris in AMD is contestable, it remains one of regions suspected to play a role in this disorder [24]. Toxic metals could be preferentially deposited in the retina via the choriocapillaris because its capillaries have fenestrated endothelium through which circulating metals can readily pass [30].

Iron and nickel were concentrated in the prelaminar and lamina cribrosa segments of the optic nerve head in some of our samples. These regions have previously been shown to take up mercury in adult and fetal monkey optic nerve head capillaries, glial cells, axons, and the connective tissue of the lamina cribrosa [17, 37]. The optic nerve head and intracranial optic nerve of fetal mice also take up transplacental mercury [35]. Tissue mercury can provoke an autoimmune response [46], so it has been suggested that a preferential uptake of mercury in the optic nerve may be one reason inflammatory demyelination in the optic nerve is common in multiple sclerosis [35]. The lamina cribrosa allows retinal ganglion cell axons and the retinal vein to leave the eye, the central retinal artery to enter the eye, and stabilises intraocular pressure by forming a barrier between the intraocular and extraocular spaces [47]. It is not clear if an accumulation of toxic metals in the lamina cribrosa would affect any of these functions. The reason for the marked uptake of metals in the optic nerve head is not know, but may be because of its rich blood supply [48], or that the metals have a predilection for unmyelinated axons [17].

**Fig 5:**
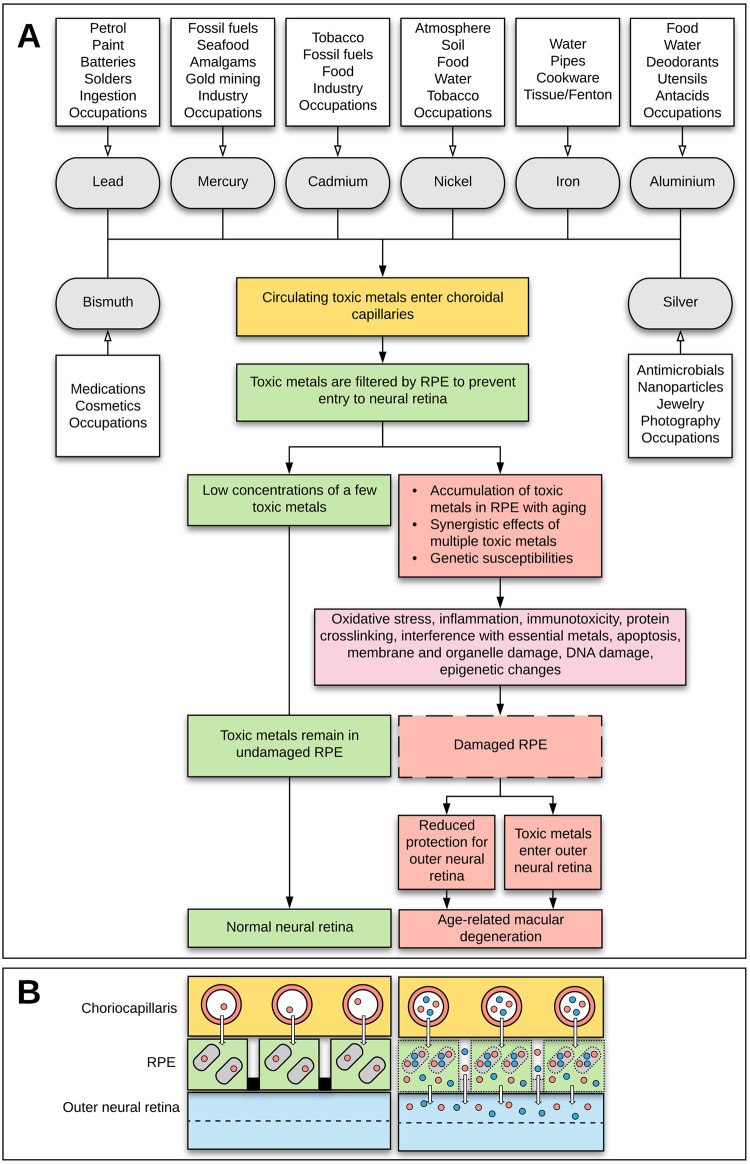
Hypothesis of toxic metal-induced age-related macular degeneration. (**A**) Exposure to a variety of toxic metals results in these metals entering capillaries of the choriocapillaris. Toxic metals are normally filtered by the retinal pigment epithelium (RPE) to prevent them entering the neural retina. The RPE is overwhelmed by toxic metals when these accumulate during aging. Synergistic effects between different toxic metals, and genetic susceptibilities, augment these toxic effects. Damage to the RPE is via multiple mechanisms, chiefly oxidative stress. Injury to RPE cells reduces its varied functions protecting the outer neural retina, and allows the entry of toxic metals into the outer neural retina, with subsequent age-related macular degeneration. (**B**) Toxic metals can enter the outer neural retina by transcellular and paracellular routes. *Left*: When the concentrations of a few circulating toxic metals (red circles in choriocapillaris capillaries) are low, they are filtered by binding to RPE melanosomes (grey rounded rectangles), and prevented from entry to the neural retina by apical tight junctions (black boxes). *Right*: With high levels of multiple toxic metals (red and blue dots in choriocapillaris capillaries), damaged melanosomes allow freed toxic metals to pass through the cell. Damaged RPE tight junctions allow toxic metals to pass between the cells, into the outer neural retina, contributing to AMD.

A pathway for the role that toxic metals may play in AMD is presented in Fig 5. The toxic metals found in our eye samples are those to which humans are commonly exposed, either from occupations, atmospheric pollution from industrial activity, burning coal and oil, certain diets, or smoking and other habits [19, 22, 49, 50]. The tight junctions of RPE cells form the outer blood-retinal barrier [51] which separates the outer retinal cells from the fenestrated capillaries in the choriocapillaris. One function of the RPE is to act as a filter for circulating toxicants entering the outer neural retina from the choriocapillaris, by binding to melanosomes within RPE cells [3, 5, 52, 53]. Other functions include transcellular and paracellular transport to supply nutrients to the retina, the removal of toxic agents from the retina, protection against photo-oxidation, and the secretion of growth factors [2, 54]. The toxic metal hypothesis for AMD postulates that throughout life, continuous or episodic exposure to toxic metals results in their accumulation in the RPE, where their damaging mechanisms are augmented by metal-metal interactions, genetic susceptibilities and lifestyle factors.

Four of our seven donors had histories of cancer, which raises the possibility that toxic metals could accumulate both in organs later affected by cancer and in the retina. Toxic metals such as mercury, cadmium and nickel, which were found often in our retinal samples, are known genotoxins that have been implicated in the pathogenesis of several tumours such as breast cancer [55–57]. Furthermore, epidemiological studies have reported associations between AMD and tumours of the lung, kidney, prostate and thyroid [58–61]. An underlying factor linking AMD and neoplasms may therefore be exposure to toxic metals, though large studies of toxic metals in people with and without both AMD and tumours would be needed to establish any such linkage.

This study has several limitations, most of which could be overcome by future studies. (**1**) No details of donors were available concerning occupation, place of residence, smoking and other personal habits, seafood consumption, amalgam fillings, and medications. We were therefore unable to establish environmental sources of the toxic metals detected in the eyes. A future study using tissue from prospectively consented participants where these data are available would be of interest. (**2**) The anonymised donor samples precluded genomic analyses to look for genetic susceptibilities. Future studies of prospectively consented participants would be needed to compare genome analyses with the toxic metals found. (**3**) Some metals such as iron are carried in red blood cells, so some of the metals detected in the choriocapillaris could have been in circulating red blood cells, rather than in tissue cells. It is likely, however, that most of the metals were in tissues, since LA-ICP-MS-detected metals were seen in the optic nerve head, which contained virtually no red blood cells (S1–7 Figs). Furthermore, a previous study showed that people with toxic metals in their eyes had only trace blood levels of these metals, suggesting that the metals had been taken up over time by the eye tissue [5]. The exact cellular location of multiple toxic levels in the eye could be determined in future x-ray microanalysis studies (which have shown, eg, iron in RPE melanosomes) when fresh frozen sections of eyes are available [3]. (**4**) We limited the number of metals analysed with LA-ICP-MS to eleven so as to ensure image fidelity and to maintain detection at low concentrations [62]. Other toxic metals suspected to play a role in AMD, such as arsenic, could be analysed in future LA-ICP-MS studies to look for a wider range of toxic metals in the human eye. (**5**) We had no eye samples from people aged less than 54 years, so we could not assess at what age toxic metals first become detectable in normal human eyes. This is worth future study, given that animal experiments indicate toxic metals can enter the eye prenatally [35, 37]. (**6**) A larger sample size of donors with no visual impairment would be valuable as a future undertaking, but we had no available cohort of clinically-validated normal aged eyes on which these experiments could be undertaken. To exclude functional or other deficits, donors would need to be prospectively consented, undergo regular detailed ophthalmic examination, including multimodal retinal and choroidal imaging, visual field studies and electroretinograms before death and eye donation. (**7**) The current study was an attempt to establish a baseline for heavy metal accumulation in normal aged human eye tissue. To further test our hypothesis, future studies would need to include eyes with age-related abnormalities at the interface of the RPE and choriocapillaris as well as those with early and advanced AMD. (**8**) Only the posterior globes were available for examination, so we were unable to look for evidence of anterior segment pathology such as that seen acute glaucoma. Longstanding glaucoma, on the other hand, would be reflected in the posterior segment by atrophy of macular ganglion cell density as well as optic nerve atrophy, neither of which were present in our study cohort.

In conclusion, we have located several toxic metals, often co-existing in the same eye, in the intact retina and optic nerve head of adult eyes. This supports the hypothesis that if sufficient quantities of these metals accumulate in the retina they could damage the retinal pigment epithelium and thereby contribute to the pathogenesis of age-related macular degeneration.

## Supporting information

S1 Fig Histological appearance of the retina and optic nerve head of seven donors. No histological abnormalities are seen in the retina or optic nerve head of any of the seven samples. Bruch’s membrane is the thin pale membrane between the retinal pigment epithelium and the choriocapillaris. AMG: autometallography, RPE: retinal pigment epithelium, R: donor identification number.
(TIF)

S2 Fig Histological appearance of the retina and optic nerve head of seven donors. No histological abnormalities are seen in the retina or optic nerve head of any of the seven samples. Bruch’s membrane is the thin pale membrane between the retinal pigment epithelium and the choriocapillaris. AMG: autometallography, RPE: retinal pigment epithelium, R: donor identification number.
(TIF)

S3 Fig Histological appearance of the retina and optic nerve head of seven donors. No histological abnormalities are seen in the retina or optic nerve head of any of the seven samples. Bruch’s membrane is the thin pale membrane between the retinal pigment epithelium and the choriocapillaris. AMG: autometallography, RPE: retinal pigment epithelium, R: donor identification number.
(TIF)

S4 Fig Histological appearance of the retina and optic nerve head of seven donors. No histological abnormalities are seen in the retina or optic nerve head of any of the seven samples. Bruch’s membrane is the thin pale membrane between the retinal pigment epithelium and the choriocapillaris. AMG: autometallography, RPE: retinal pigment epithelium, R: donor identification number.
(TIF)

S5 Fig Histological appearance of the retina and optic nerve head of seven donors. No histological abnormalities are seen in the retina or optic nerve head of any of the seven samples. Bruch’s membrane is the thin pale membrane between the retinal pigment epithelium and the choriocapillaris. AMG: autometallography, RPE: retinal pigment epithelium, R: donor identification number.
(TIF)

S6 Fig Histological appearance of the retina and optic nerve head of seven donors. No histological abnormalities are seen in the retina or optic nerve head of any of the seven samples. Bruch’s membrane is the thin pale membrane between the retinal pigment epithelium and the choriocapillaris. AMG: autometallography, RPE: retinal pigment epithelium, R: donor identification number.
(TIF)

S7 Fig Histological appearance of the retina and optic nerve head of seven donors. No histological abnormalities are seen in the retina or optic nerve head of any of the seven samples. Bruch’s membrane is the thin pale membrane between the retinal pigment epithelium and the choriocapillaris. AMG: autometallography, RPE: retinal pigment epithelium, R: donor identification number.
(TIF)
