# Supplementary figures and images for: The distribution of toxic metals in the human retina and optic nerve head: Implications for age-related macular degeneration
Source: PLoS One. 2020 Oct 29;15(10):e0241054. doi: 10.1371/journal.pone.0241054 (PMC7595417; doi:10.1371/journal.pone.0241054)

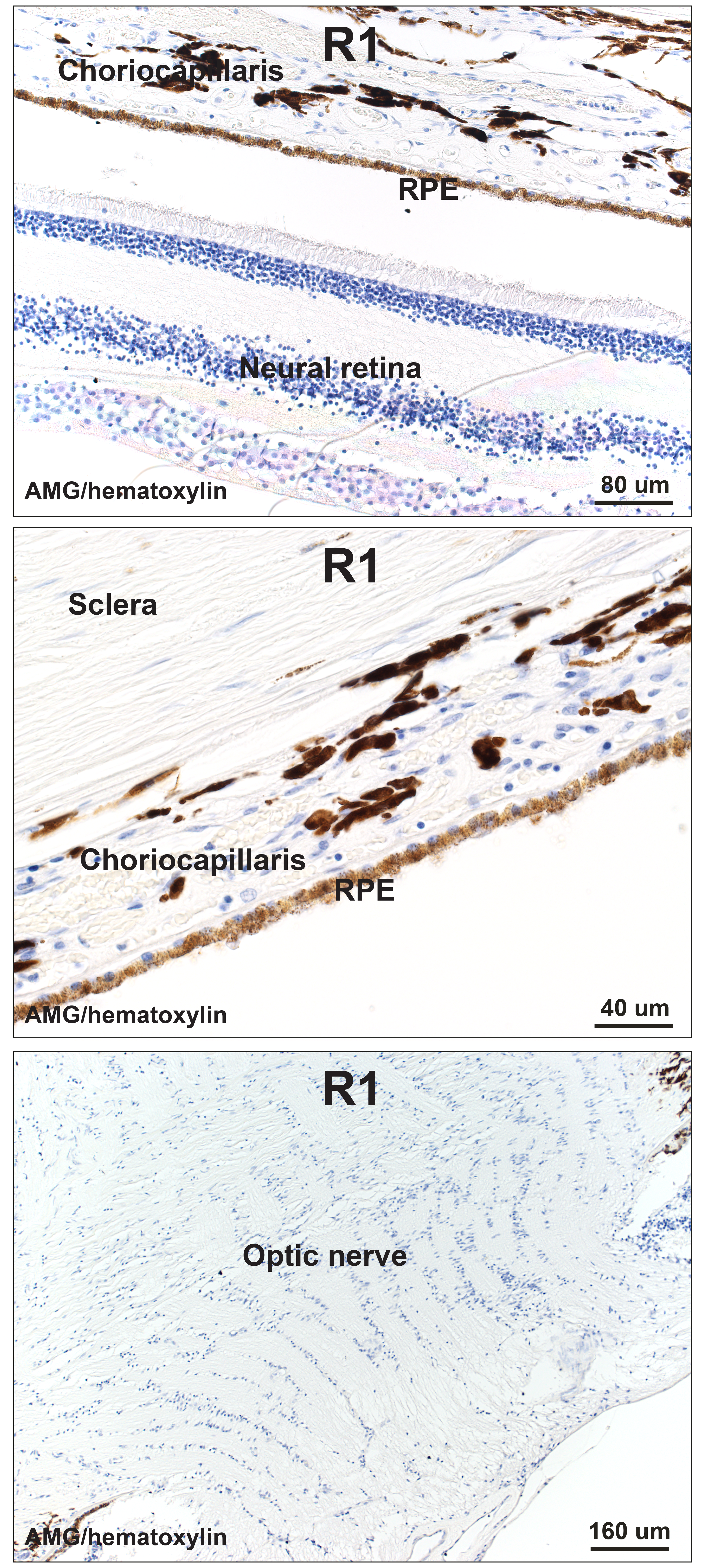

Supplement: S1 Fig — No histological abnormalities are seen in the retina or optic nerve head of any of the seven samples. Bruch’s membrane is the thin pale membrane between the retinal pigment epithelium and the choriocapillaris. AMG: autometallography, RPE: retinal pigment epithelium, R: donor identification number. (TIF) [file pone.0241054.s001.tif]

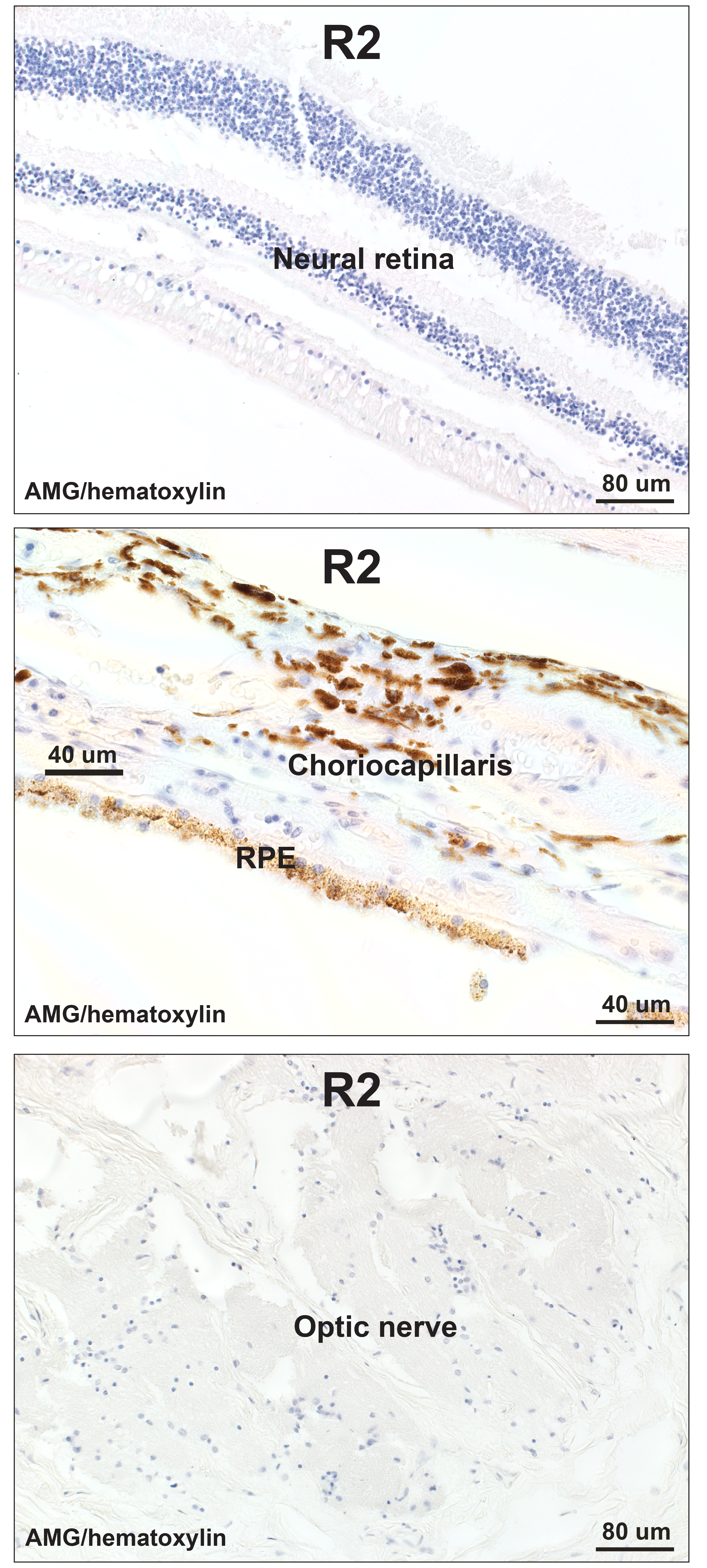

Supplement: S2 Fig — No histological abnormalities are seen in the retina or optic nerve head of any of the seven samples. Bruch’s membrane is the thin pale membrane between the retinal pigment epithelium and the choriocapillaris. AMG: autometallography, RPE: retinal pigment epithelium, R: donor identification number. (TIF) [file pone.0241054.s002.tif]

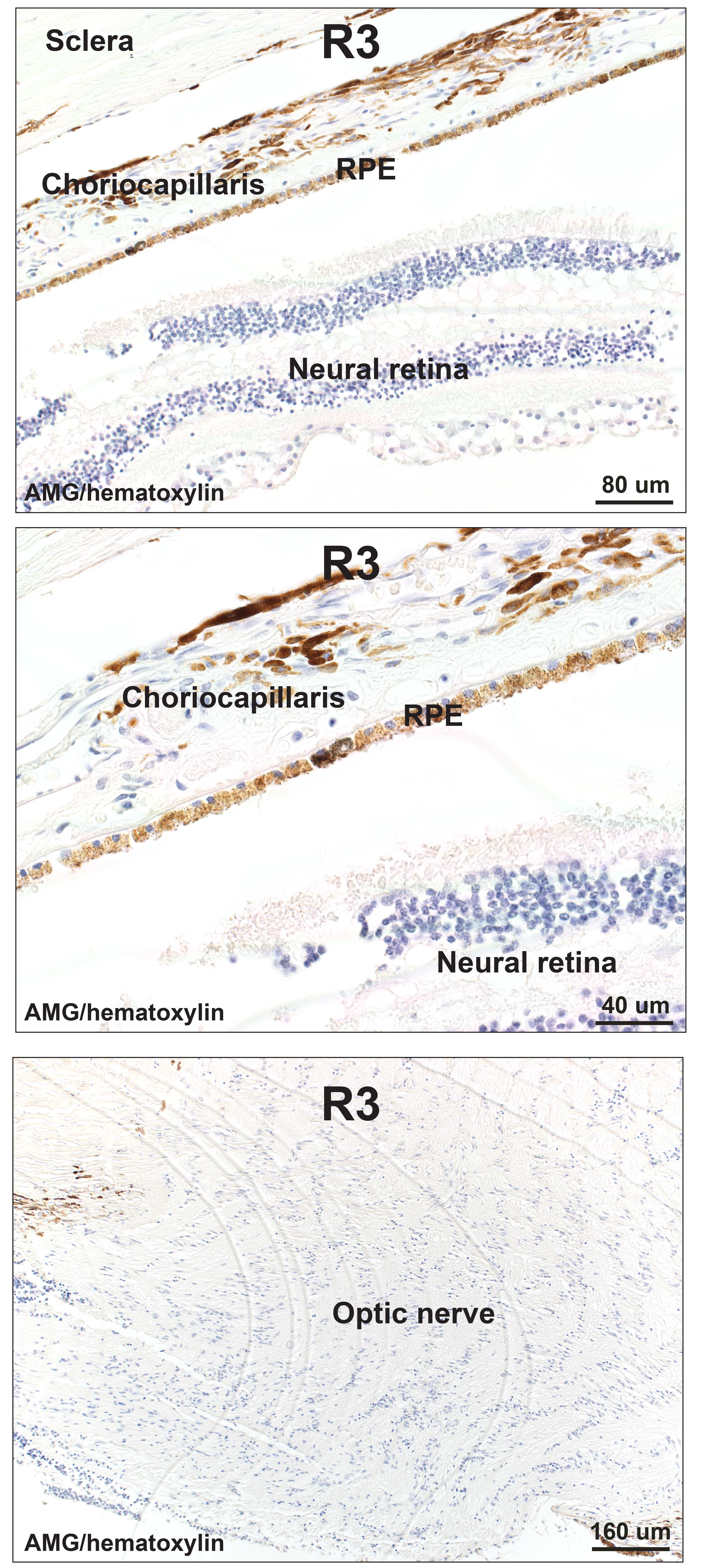

Supplement: S3 Fig — No histological abnormalities are seen in the retina or optic nerve head of any of the seven samples. Bruch’s membrane is the thin pale membrane between the retinal pigment epithelium and the choriocapillaris. AMG: autometallography, RPE: retinal pigment epithelium, R: donor identification number. (TIF) [file pone.0241054.s003.tif]

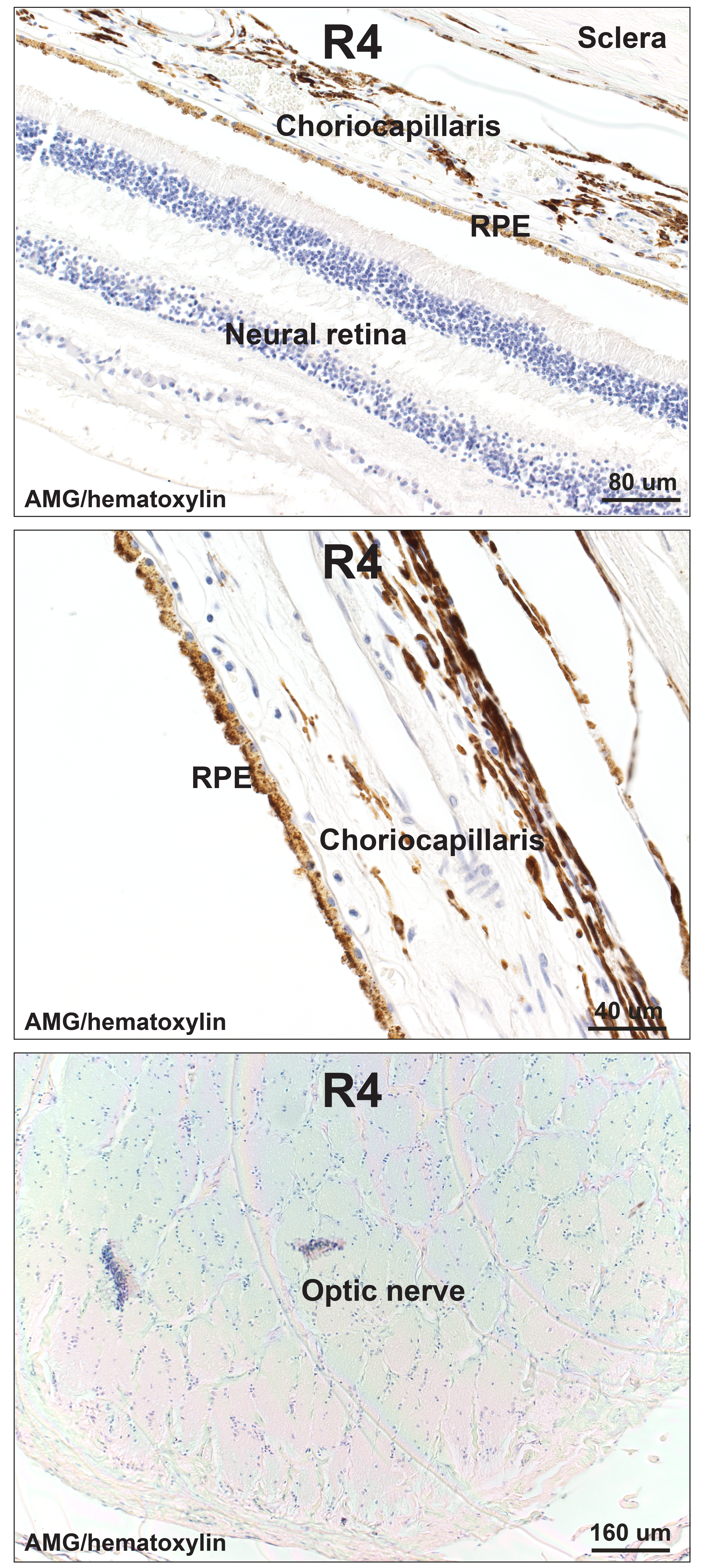

Supplement: S4 Fig — No histological abnormalities are seen in the retina or optic nerve head of any of the seven samples. Bruch’s membrane is the thin pale membrane between the retinal pigment epithelium and the choriocapillaris. AMG: autometallography, RPE: retinal pigment epithelium, R: donor identification number. (TIF) [file pone.0241054.s004.tif]

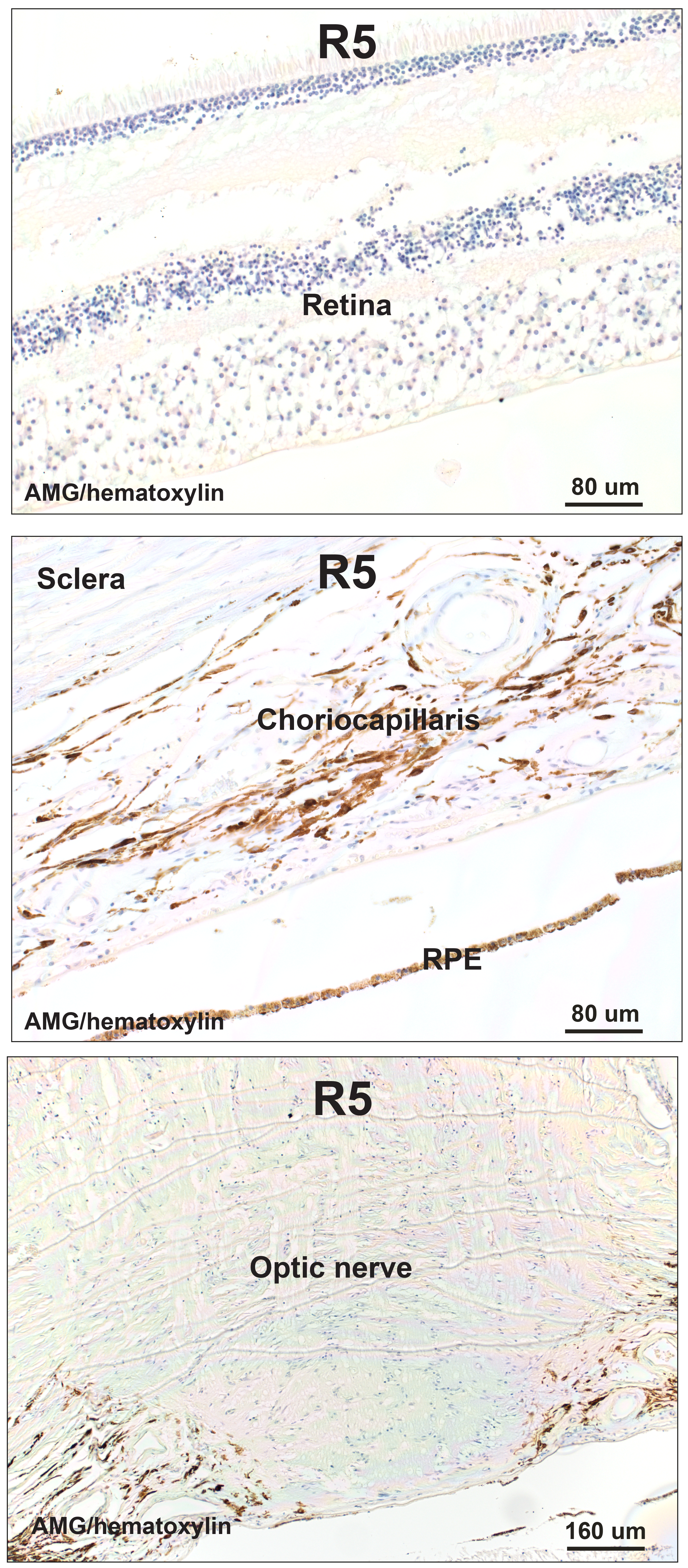

Supplement: S5 Fig — No histological abnormalities are seen in the retina or optic nerve head of any of the seven samples. Bruch’s membrane is the thin pale membrane between the retinal pigment epithelium and the choriocapillaris. AMG: autometallography, RPE: retinal pigment epithelium, R: donor identification number. (TIF) [file pone.0241054.s005.tif]

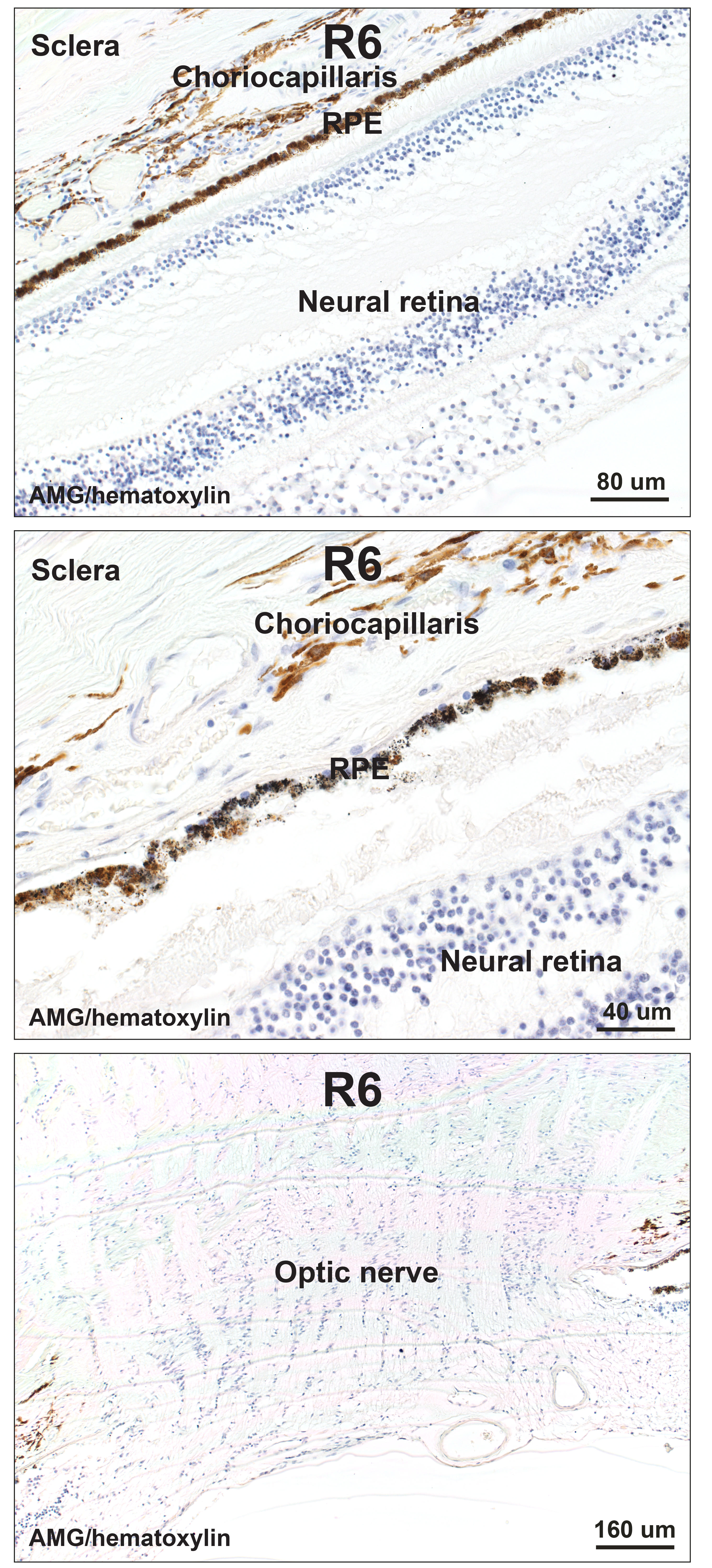

Supplement: S6 Fig — No histological abnormalities are seen in the retina or optic nerve head of any of the seven samples. Bruch’s membrane is the thin pale membrane between the retinal pigment epithelium and the choriocapillaris. AMG: autometallography, RPE: retinal pigment epithelium, R: donor identification number. (TIF) [file pone.0241054.s006.tif]

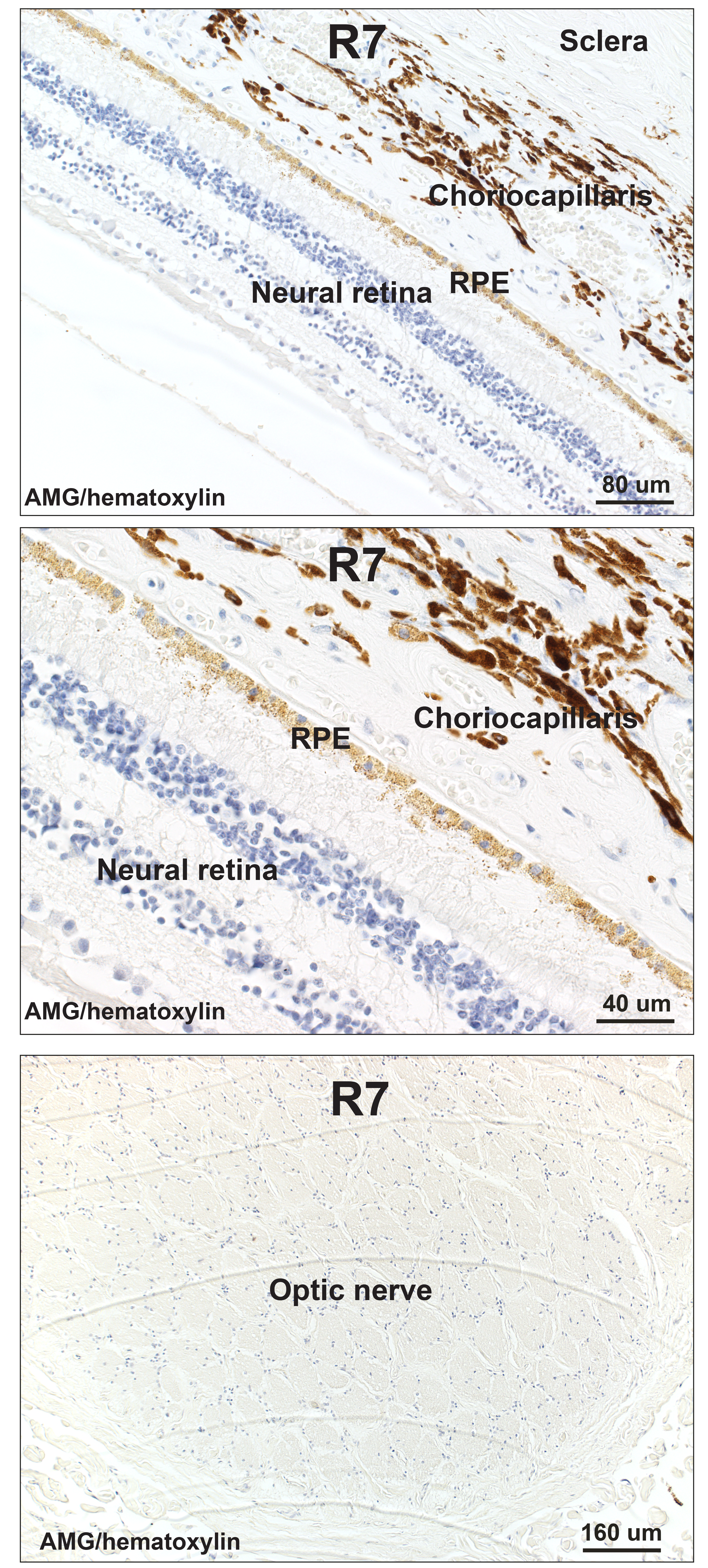

Supplement: S7 Fig — No histological abnormalities are seen in the retina or optic nerve head of any of the seven samples. Bruch’s membrane is the thin pale membrane between the retinal pigment epithelium and the choriocapillaris. AMG: autometallography, RPE: retinal pigment epithelium, R: donor identification number. (TIF) [file pone.0241054.s007.tif]
